# Simulation of forced convection in a channel with nanofluid by the lattice Boltzmann method

**DOI:** 10.1186/1556-276X-8-178

**Published:** 2013-04-17

**Authors:** Nor Azwadi Che Sidik, Maysam Khakbaz, Leila Jahanshaloo, Syahrullail Samion, Amer Nordin Darus

**Affiliations:** 1 Faculty of Mechanical Engineering, Universiti Teknologi Malaysia, UTM Skudai, Johor, 81310, Malaysia

**Keywords:** Nanofluid, Volume fraction, Nusselt number, Reynolds number, Lattice Boltzmann method

## Abstract

This paper presents a numerical study of the thermal performance of fins mounted on the bottom wall of a horizontal channel and cooled with either pure water or an Al_2_O_3_-water nanofluid. The bottom wall of the channel is heated at a constant temperature and cooled by mixed convection of laminar flow at a relatively low temperature. The results of the numerical simulation indicate that the heat transfer rate of fins is significantly affected by the Reynolds number (Re) and the thermal conductivity of the fins. The influence of the solid volume fraction on the increase of heat transfer is more noticeable at higher values of the Re.

## Background

Recent years have eyewitnessed a blossom flourishing in the evolvement of electronics, communications, and auto-computing industries, and this bearing is irrefutably continuing in this century. The cooling of electrical, mechanical, and electronic components has become troublesome in today's fast-growing technologies. Inasmuch as the significance of heat exchangers in tremendous engineering applications, the subject of potential heat transfer enhancement in these devices has received sizeable attention in practice and research. On account of the fact that the consistency of the electronic components commodiously increases, conspicuous lack of heat transfer enhancement both in macro- and microscales of channels is realized. Encountering a fluid flow by utilizing transverse surfaces in a channel is a prevalent method that is used to intensify the rate of heat transfer from heated surfaces.

Alamyane and Mohamad [1] studied the forced convection heat transfer in a channel with extended surfaces. The effects of the Reynolds number (Re) and the fin height and spacing on the fluid flow and the heat transfer were examined. Yang et al. [2] simulated the forced convection in a parallel plate channel. Constant temperature was considered in both upper and lower walls, and a transverse object was located at the lower channel wall. The effects of the Reynolds number, the thermal conductivity ratio of the fluid, and the fin profile area on the fluid flow and the heat transfer rate were analyzed. The study results showed that the heat transfer enhancement with an increment of the Reynolds number and the thermal conductivity ratio of the fluid at various fin profiles. Yang et al. [3] numerically investigated the effect of mix convection heat transfer in an inclined parallel plate channel with a transverse object at the bottom wall. In this research, the effects of thermal conductivity, Reynolds number, the fin profile, and the channel inclination on the heat transfer rate at various Richardson numbers were examined. They discovered that the ace aspect ratio of the fin was related to the fin with utmost heat transfer at various Reynolds and Richardson numbers.

Young and Vafai [4] observed the impact of controlling parameters on the cooling of heated channels with mounted objects. Concentrating on the effect of altering the dimensions of the object, the thermal conductivity, the heating method, and the Re was embraced. They deduced that the fluid flow and heat transfer are affected by the geometry and material of the object, and a correlation for the average Nusselt number was proposed as a function of the controlling parameters.

Meinders and Hanjalic [5] experimentally investigated the effect of the cubes' arrangement on the turbulent fluid flow. They comprehended that the flow stream was affected by the distance between the objects owing to the fact of augmenting the flow velocity. Moreover, amelioration in velocity distribution and heat transfer than the staggered distribution case was found for flow over inline cubes. Yan et al. [6] experimentally investigated the influence of short surface-mounted objects at the top of a flat plate on the heat transfer enhancement. Scrutinizing was done on the effect of varies cross sections, spacing and numbers of objects, and the Reynolds number. They perceived that the heat transfer was incremented when the height of the object is comparatively equal to half of the channel height.

In an experimental investigation by Yuan et al. [7], the heat transfer and friction characteristics of a channel which were attached by winglets were examined. Heat transfer from the channel was achieved to be noticeably augmented by using winglets in comparison with conventional channels with rectangular transverse objects. For a high Reynolds number, the heat transfer was enhanced by a factor of 2.7 to 6 times of the smooth channel.

Utilizing nanofluids for the purpose of enhancing the heat transfer in thermal systems is another alternative technique [8]. The thermal performance of different types of nanofluids has been the subject of many recent studies on forced, natural, and mixed convection problems. Several explorations have studied natural convection of nanofluids in cavities [9,10]. They argued that the addition of nanoparticles in the fluid indisputably increase the natural convection heat transfer.

Chein and Huang [11] analyzed the cooling of two silicon microchannel heat sinks with a water-Cu nanofluid. The heat transfer and fraction coefficients were based on the theoretical models and the experimental correlations. They realized that the heat transfer performance of microchannels was greatly improved when nanofluids were added into base fluid as coolants without any extra pressure drop.

Recently, Santra et al. [12] numerically investigated the effect of water-Cu nanofluid through parallel plate channel in laminar forced convection. A cold nanofluid was sent through the channel, and the walls of the channel were isothermally heated. The effects of the Reynolds number and the solid volume fraction on the heat transfer were studied by considering the fluid to be Newtonian and non-Newtonian. They observed that the rate of heat transfer increased with an increase of the Reynolds number and the solid volume fraction. The increase in the heat transfer was approximately the same for both scenarios.

The lattice Boltzmann method (LBM) is another numerical method that is often used to simulate flow problems. LBM have been used for more than 2 decades as an alternative numerical technique. In LBM, it is intended to model fluids as a collection of particles, which successively undergo collision and propagation over a discrete lattice mesh. Several lattice Boltzmann models have been proposed for the incompressible Navier–Stokes equations. A collision model was proposed by Bhatnagar et al. [13] to simplify the analysis of the lattice Boltzmann equation, which leads to the so-called lattice BGK model. Remarkable efforts have been conducted by many researchers that made this numerical method more attractive for fluid dynamics modeling, e.g., [14,15]. For more details about LBM and its application, kindly refer to the aforementioned publications.

Most of the researches cited above considered the heat transfer enhancement by adding either the fin or using nanofluids. The main objective of this study is to examine both of these effects on the heat transfer performance. In general, previous works were performed to investigate different cases of nanofluid flow and heat transfer in channels with mounted objects by focusing on changing geometries, arrangement, and dimensions of the objects. However, more efforts are needed in order to optimize the controlling parameters for best heat transfer enhancement.

## Methods

### Problem definition

**Figure 1:**
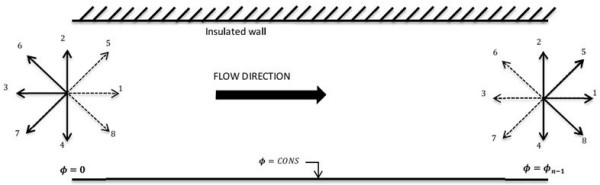
A schematic plot of flow in a channel.

The geometry of the problem is shown in Figure 1. A cold mixture of base fluid (water) and the nanoparticles (alumina) is forced to flow into a channel that is heated from its bottom and kept at a constant high temperature, while the top wall is insulated. The channel aspect ratio is fixed at L/H = 15. The Prandtl number is taken as 7.02, and the Reynolds numbers are 10, 50, and 100, whereas the extended surfaces' height to space ratio l/S is 0.2, and the ratio between the objects' height to the channel's height l/H is 0.2.

The flow is assumed as Newtonian, laminar, two-dimensional, and incompressible. In addition, it is assumed that the cold mixture of base fluid (water) and the solid spherical nanoparticles (alumina) is in thermal equilibrium, and it flows at the same velocity as a homogenous mixture.

### Numerical simulation

**Figure 2:**
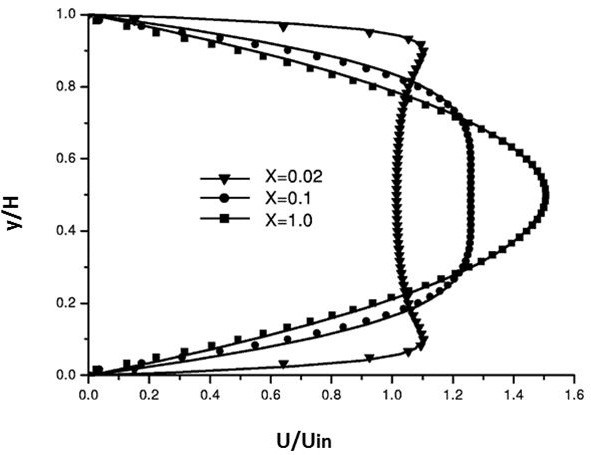
A schematic plot showing the thermal boundary conditions of the problem.

The D2Q9 LBM model is used to simulate fluid flow in two-dimensional channel with uniform grid size of *δx* × *δy*. The lattice Boltzmann equation (known as LBGK equation) with single relaxation time can be expressed as [13]

(1) $$f_{i}\left(x+e_{i}\Delta t,t+\Delta t\right)-f_{i}\left(x,t\right)=-\frac{1}{\tau_{f}}\left(f_{i}-f_{i}^{\mathrm{eq}}\right)$$

which can be reformulated as

(2) $$f_{i}\left(x+e_{i}\Delta t,t+\Delta t\right)=\omega_{f}f_{i}^{\mathrm{eq}}+\left[1-\omega_{f}\right]f_{i}$$

where $\omega_{f}=\frac{1}{\tau_{f}}$ and *τ*_*f*_ as the single relaxation time of the fluid, *f*_*i*_ represents the particle distribution function, **e**_*i*_ is the particle streaming velocity, and $f_{i}^{\mathrm{eq}}$ is the local equilibrium distribution function. For D2Q9 model $f_{i}^{\mathrm{eq}}$ is given by [8]

(3) $$f_{i}^{\mathrm{eq}}=\rho \omega_{i}\left[1+3e_{i}\cdot u+\frac{9}{2}{\left(e_{i}\cdot u\right)}^{2}-\frac{3}{2}u^{2}\right]$$

where *ρ* is the density of the fluid and *ω*_*i*_ is the weight function, which has the values of $\omega_{0}=\frac{4}{9}$, $\omega_{i}=\frac{1}{9}$ for _*i*_ = 1 to 4, and $\omega_{i}=\frac{1}{36}$ for *i* = 5 to 8. The macroscopic fluid flow velocity in lattice units is represented by **u**. In the LBM, the fluid macroscopic quantities such as density, *ρ*, and flow momentum, *ρ***u** are calculated using the distribution function *f*_*i*_, and given by $\rho =\overset{8}{\underset{i=0}{\sum }}f_{i}$ and $\rho u=\overset{8}{\underset{i=0}{\sum }}e_{i}f_{i}$, respectively. The streaming speed for particles in coordinate (*x* and *y*) directions (i.e., 1 to 4, see Figure 2) can be expressed as **e**_*i*_ = cos(*π*/2 (*i* − 1)), sin(*π*/2 (*i* − 1)), whereas particles in diagonal directions (i.e., 5 to 8 in Figure 2) have velocities of $e_{i}=\sqrt{2}\left(\cos\left(\pi /4\left(2i-9\right)\right),\sin\left(\pi /4\left(2i-9\right)\right)\right)$; however, the particle in the lattice center is at rest and has no streaming speed, i.e., **e**_0_ = 0.

The thermal part is simulated using another distribution function for the temperature. For instance, *g* is used to simulate the distribution function of the dependent variable (temperature) in the lattice Boltzmann equation, and an approach similar to that used to simulate the fluid flow is utilized to simulate the temperature distribution. In addition, the algorithm suggested by Succi [15] is adopted throughout this work. The kinetic equation for the temperature distribution function with single relaxation time is given by:

(4) $$g_{i}\left(x+e_{i}\Delta t,t+\Delta t\right)-g_{i}\left(x,t\right)=-\frac{1}{\tau_{t}}\left(g_{i}-g_{i}^{\mathrm{eq}}\right)$$

which can be written in the form

(5) $$g_{i}\left(x+e_{i}\Delta t,t+\Delta t\right)=\omega_{t}g_{i}^{\mathrm{eq}}+\left[1-\omega_{t}\right]g_{i}$$

Where *g*_*i*_ represents the temperature distribution function of the particles, $g_{i}^{\mathrm{eq}}$ is the local equilibrium distribution function of the temperature, and $\omega_{t}=\frac{1}{\tau_{t}}$, where *τ*_*t*_ is the single relaxation time of the temperature distribution. Thus, the equilibrium distribution function of the thermal part is given by [15]:

(6) $$g_{i}^{\mathrm{eq}}=\phi \left(x,t\right)\omega_{i}\left[1+\frac{e_{i}\cdot u}{C_{s}^{2}}\right]$$

where, *ϕ* is the macroscopic temperature and $C_{s}^{2}$ is the speed of sound. The diffusion coefficient can be obtained as a function of the relaxation time and given by $\frac{\Delta x^{2}}{\Delta t}\left(\frac{1}{\omega }-\frac{1}{2}\right)$. The macroscopic temperature is then computed from:

(7) $$\phi \left(x,t\right)=\overset{8}{\underset{i=0}{\sum }}g_{i}=\overset{8}{\underset{i=0}{\sum }}g_{i}^{\mathrm{eq}}$$

A uniform lattice of 100 × 1,500 is used to perform all of the simulations. However, the number of lattices was doubled to test the grid dependency results.

Since the inlet velocity of the flow is specified, the inward distribution functions should be computed at the boundary. In the D2Q9 model, the values of the distribution functions pointing out of the domain at the inlet boundary (i.e., *f*_3_, *f*_6_, *f*_7_ in Figure 2) are known from the streaming step, and the only unknowns are (*f*_1_, *f*_5_, *f*_8_) as well as the fluid density *ρ*. Following the work of Zou and He [16], the inlet density and the distribution functions can be obtained from:

(8) $$\rho_{\mathrm{in}}=\frac{1}{1-u_{\mathrm{in}}}\left[f_{0}+f_{2}+f_{4}+2\left(f_{3}+f_{6}+f_{7}\right)\right]$$

The unknown distribution functions are calculated using

(9) $$\begin{array}{l} f_{1}=f_{3}+\frac{2}{3}\rho u_{x}\\ f_{5}=f_{7}-\frac{1}{2}\left(f_{2}-f_{4}\right)+\frac{1}{6}\rho u_{x}+\frac{1}{2}\rho u_{y}\\ f_{8}=f_{6}+\frac{1}{2}\left(f_{2}-f_{4}\right)+\frac{1}{6}\rho u_{x}+\frac{1}{2}\rho u_{y} \end{array}$$

An extrapolation scheme is used to simulate the outlet flow condition, which can be represented as *f*_*i*_(*N*_*x*_, *t*) = *f*_*i*_(*N*_*x*_ − 1, *t*), *i* = 3, 6, 7. The bounce-back scheme is used to specify the boundary conditions on solid surfaces (no-slip boundary), in which the distribution functions pointing to the fluid are equal to those pointing out of the domain. The thermal boundary conditions for this case are given in Figure 2. For constant wall temperature (the lower wall temperature is constant), the unknown functions are obtained using the following equation [15]:

(10) $$g_{i}=\phi_{\mathrm{wall}}\left(\omega_{i}+\omega_{i+2}\right)-g_{i+2}\;\mathrm{for}\;i=2,5\;\mathrm{and}\;6$$

The left-hand boundary (channel inlet) is kept at a constant temperature (Dirichlet boundary condition) and set to a dimensionless value of zero. Therefore, the resulted equations of the unknown distribution functions on the left boundary are given by *g*_*i*_(0, *t*) = − *g*_*i* + 2_(0, *t*), for *i* = 1, 5, and 8. For the adiabatic boundary condition, the gradient of the dependent variable normal to the boundary should be zero, i.e., ∂ *φ*/∂ *y* = 0. The distribution functions are found to be in the following form [15]:

(11) $$\overset{8}{\underset{i=0}{\sum }}g_{i}\left(x,N_{y}+1\right)=\overset{8}{\underset{i=0}{\sum }}g_{i}\left(x,N_{y}\right)$$

A second-order extrapolation similar to the one given in [17] is used to obtain the values of the unknown distribution functions for the right-hand side boundary (channel outlet) as follows:

(12) $$\begin{array}{l} g_{i}\left(N_{x},t\right)=2g_{i}\left(N_{x}-1,t\right)-g_{i}\left(N_{x}-2,t\right)\mathrm{for}\;i\\ \;=3,6,\mathrm{and}\;7. \end{array}$$

The local Nusselt number (*Nu*_*x*_) is computed using the following equation:

(13) $$Nu_{x}=\frac{L_{C}{\left(\partial \phi /\partial y\right)}_{y=0}}{\phi_{\mathrm{wall}}-\phi_{m}}$$

where *L*_*c*_ is the characteristic length and *ϕ*_*wall*_ is the wall constant temperature. The mean temperature *ϕ*_*m*_ is given by:

(14) $$\phi_{m}=\frac{\int \mathrm{u\phi dA}}{\int \mathrm{udA}}$$

The effective density of the nanofluid is

(15) $$\rho_{\mathrm{nf}}=\left(1-\phi \right)\rho_{f}+\phi \rho_{s}$$

where *ϕ* is the solid volume fraction. The effective dynamic viscosity of the nanofluid given by Brinkman [18] is

(16) $$\mu_{\mathrm{nf}}=\frac{\mu_{f}}{{\left(1-\phi \right)}^{2.5}}$$

The thermal diffusivity of the nanofluid is

(17) $$\alpha_{\mathrm{nf}}=\frac{k_{\mathrm{eff}}}{{\left(\rho c_{p}\right)}_{\mathrm{nf}}}$$

The heat capacitance of the nanofluid is

(18) $${\left(\rho c_{p}\right)}_{\mathrm{nf}}=\left(1-\phi \right){\left(\rho c_{p}\right)}_{f}+{\left(\rho c_{p}\right)}_{s}$$

*k*_*eff*_ is the effective thermal conductivity of the nanofluid and is determined using the model proposed by Patel et al. [19]. For the two-component entity of spherical particle suspension, the model gives:

(19) $$k_{\mathrm{eff}}=k_{f}\left[1+\frac{k_{s}A_{s}}{k_{f}A_{f}}+ck_{s}\mathrm{Pe}\frac{A_{s}}{k_{f}A_{f}}\right]$$

where *k*_*s*_ and *k*_*f*_ are the thermal conductivities of dispersed Al_2_O_3_ nanoparticles and pure water.

(20) $$\mathrm{Pe}=\frac{u_{s}d_{s}}{\alpha_{f}}$$

where *u*_*s*_ is the Brownian motion velocity of the nanoparticles given by:

(21) $$u_{s}=\frac{2k_{b}T}{\pi \mu_{f}d_{s}^{2}}$$

where *k*_*b*_ = 1.3087×10^−23^JK^−1^ is the Boltzmann constant.

## Results and discussion

### Code validation and computational results

**Figure 3:**
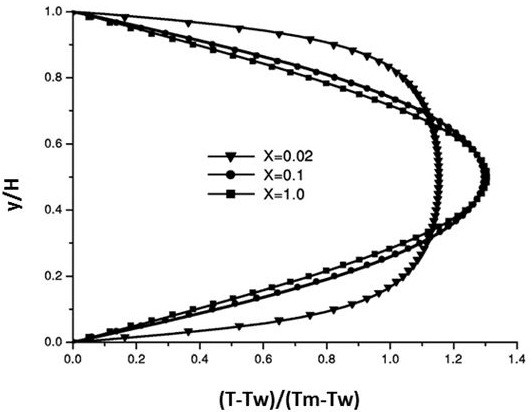
Velocity and profiles at different cross sections.

**Figure 4:**
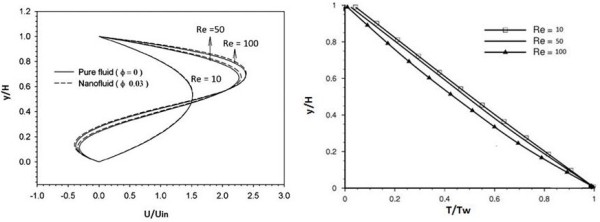
Temperature profiles at different cross sections.

For the purpose to ensure that the obtained results are proper and that the code is free of errors, a flow of cold air in a two-dimensional heated channel was taken as a benchmark test. Both upper and lower walls were heated. The comparisons were carried up between the dimensionless velocity and temperature fields at different locations in the channel as shown in Figures 3 and 4. The obtained results were found to be identical to the results of [20].

**Figure 5:**
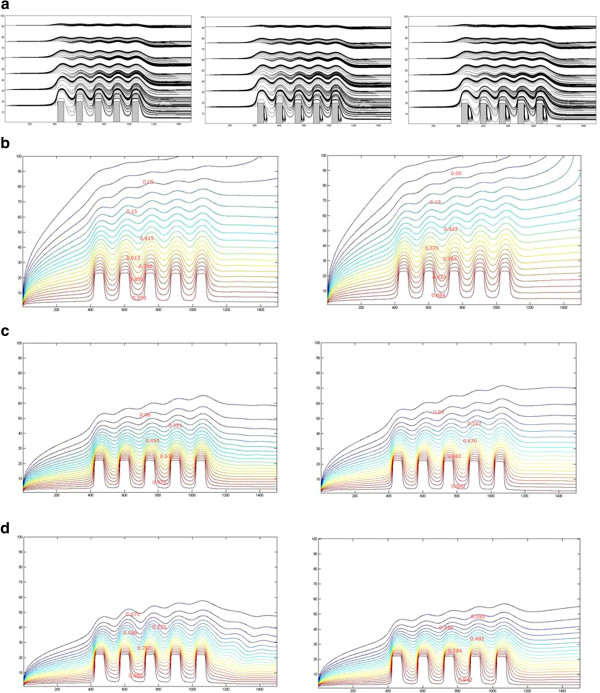
Velocity and temperature profiles at different Re.

Figure 5 shows the effect of Reynolds on the temperature profiles at the same cross sections for Re = 10, 50, and 100. The figures depicted that the temperature profiles are less sensitive to the change in Reynolds compared to the velocity profiles.

**Figure 6:**
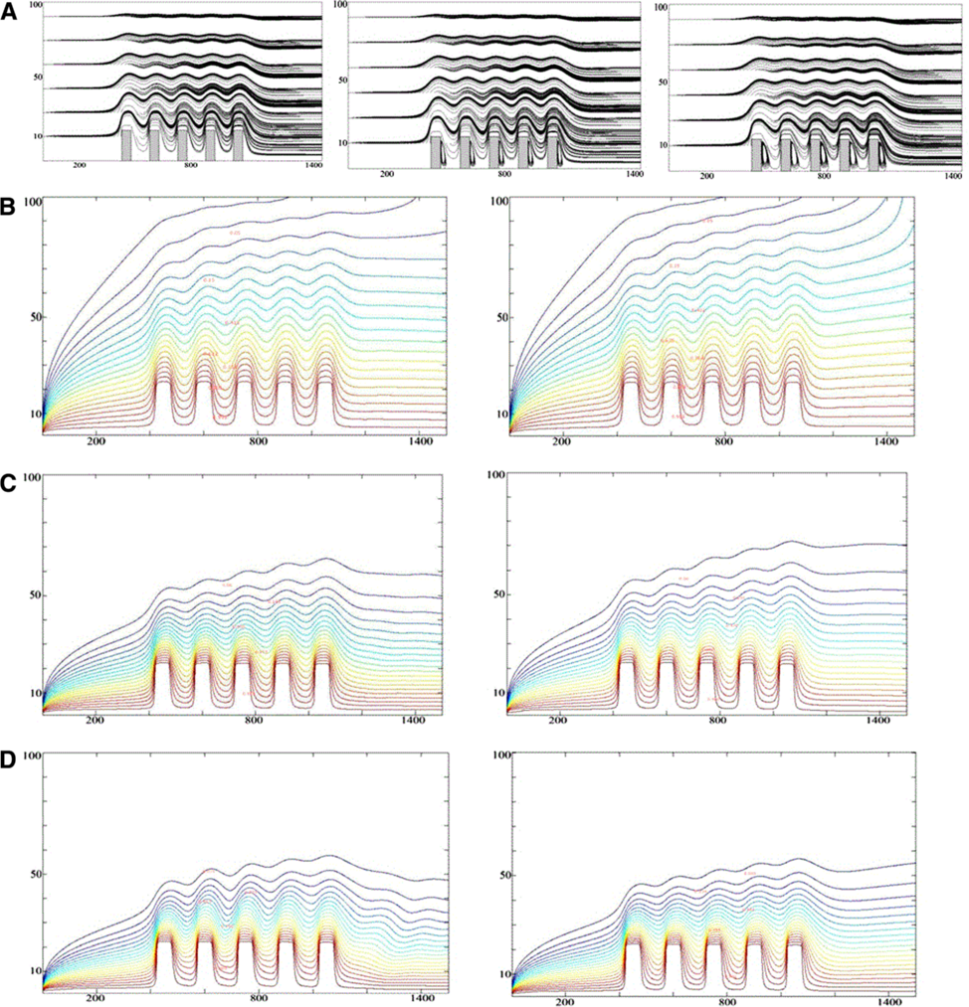
**Streamlines and isotherms for the Al**_**2**_**O**_**3**_**-water nanofluid and pure water at different Reynolds number.** (**A**) Streamline plots at (a) Re = 10, (b) Re = 50, and (c) Re = 100. (**B**) Isotherm plots at Re = 10 and (a) *φ* = 0.0 and (b) *φ* = 0.05. (**C**) Isotherm plots at Re = 50 and (a) *φ* = 0.0 and (b) *φ* = 0.05. (**D**) Isotherm plots at Re = 100 and (a) *φ* = 0.0 and (b) *φ* = 0.05.

The effects of the Reynolds number and the solid volume fraction on the heat transfer, isotherms, and streamlines are studied. Figure 6 presents the streamlines and the isotherms for the Al_2_O_3_-water nanofluid (*ϕ* = 0.05) and pure water at different Reynolds number (Re = 10, 50, and 100).

The streamlines show that as the Reynolds number increases, the vortices that are formed behind the fins become larger and stronger. This can be more clearly illustrated in Figure 5 where the horizontal velocity in the middle section between fins is presented. At Re = 10, the velocity is consistently positive. However, as the Reynolds number increases, the flow velocity becomes negative. This is an indication of flow reversal. The strong vortex at high numbers enhances the heat transfer from left face objects to right face objects and the wall between the two fins. This difference, however, becomes noticeable at higher Re.

At low Reynolds numbers, the conduction is the dominating mechanism of heat transfer. Therefore, the isotherms stretch above the fins and take a large area in the channel. As Re increases, the convection becomes the dominating mechanism, and the strong cold inlet flow pushes the isotherms near the bottom wall. The comparison between the isotherms of the nanofluid and pure water shows that in each point of the channel, the nanofluid temperature is higher than the pure water. It is due to the nanofluid's higher thermal conductivity.

**Figure 7:**
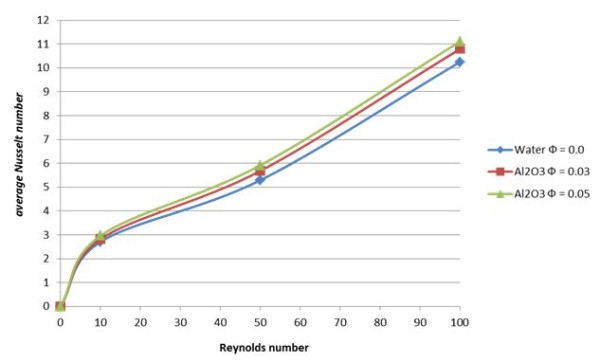
Average Nusselt number for various Re.

The current investigation is wrapped with the analysis of the effect of the Reynolds number and percentage of nanoparticle volume fraction on the heat transfer enhancement in the channel. Figure 7 and Table 1 display values of average Nusselt number at various Reynolds numbers and solid volume fraction from 0% to 5%. These figures demonstrate that the Nusselt number increases with the Reynolds number for values of volume fraction tested in the present study. For example, at Re = 100, in the addition of volume fraction of 5%, the average Nusselt number increases about 17%. High Reynolds number results in high energy transport through the fluid and cause irregular motion of nanoparticle. The higher solid volume fraction further stimulates the flow and contributes to higher Nusselt number as shown in the figure. The presence of nanoparticles also increases the rate of heat transfer by conduction mode through the flow.

**Table 1:** Average Nusselt number for various Reynolds number and solid volume fraction

**Reynolds number**	**Average Nusselt number**	***φ*= 0.0**	***φ*= 0.03**	***φ*= 0.05**
Re = 10	Nu_ave_	2.712	2.826	2.965
Re = 50	Nu_ave_	5.294	5.683	5.919
Re = 100	Nu_ave_	10.252	10.797	11.109

## Conclusions

LBM was applied to simulate forced convection heat transfer in two-dimensional channel including extended surfaces to investigate the effect of changing different parameters such as Reynolds number (10, 50, and 100) and nanofluid (Al_2_O_3_) volume fractions (0.0, 0.03, and 0.05). The results showed that as the Reynolds number increases, the rate of heat transfer also increases. The formation of vortices both in front and behind the objects enhances the heat transfer process. As the solid volume fraction increases, the heat transfer is enhanced for all values of the Reynolds numbers. This enhancement is more significant at high Reynolds numbers. The heat transfer rate of the fins increases with the thermal conductivity ratio of the fin to pure water. This enhancement has a finite limit. At this limit, the temperature at all surfaces of the fins approach the wall temperature. In this condition, the fins behave like constant temperature of heat sources.

## Competing interests

The authors declare that they have no competing interests.

## Authors’ contributions

MK, LJ, and SS conceived the study and checked the grammar of the manuscript. NACS and AND drafted the manuscript. All authors read and approved the final manuscript.
